# A Droplet Digital PCR Method for Simultaneous Detection and Quantification of *S. aureus*, *L. monocytogenes*, *C. sakazakii*, and *M. bovis* in Dairy Products

**DOI:** 10.3390/foods15132350

**Published:** 2026-07-02

**Authors:** Pengli Kong, Xiao Han, Kangdong Huang, Hong Yang, Hongfei Mo, Huan Li, Linglin Fu, Hui Qiu, Jiangbing Shuai

**Affiliations:** 1 Hangzhou Customs Technical Center, Hangzhou 311202, China; kongpengli1110@163.com (P.K.); mohf@ziq.gov.cn (H.M.); 2 Zhejiang Academy of Science and Technology for Inspection and Quarantine, Hangzhou 310016, China; hanx@zaiq.org.cn (X.H.); hkd@zaiq.org.cn (K.H.); 3 College of Animal Sciences, Zhejiang University, Hangzhou 310058, China; 4 College of Animal Science and Technology, Zhejiang Agriculture & Forestry University, Hangzhou 311300, China; 2023608041017@stu.zafu.edu.cn; 5 Food Safety Key Laboratory of Zhejiang Province, School of Food Science and Biotechnology, Zhejiang Gongshang University, Hangzhou 310018, China; huanli@mail.zjgsu.edu.cn (H.L.); fulinglin@mail.zjgsu.edu.cn (L.F.)

**Keywords:** droplet digital PCR, foodborne pathogens, dairy products, *S. aureus*, *L. monocytogenes*, *C. sakazakii*, *M. bovis*

## Abstract

Foodborne bacterial pathogens, including *S. aureus*, *L. monocytogenes*, *C. sakazakii*, and *M. bovis*, pose significant threats to dairy safety and public health. Current detection methods, such as culture-based techniques and real-time quantitative PCR (qPCR), are either time-consuming or limited in absolute quantification accuracy. Herein, we developed and validated a novel quadruplex droplet digital PCR (ddPCR) assay for simultaneous detection and absolute quantification of these four pathogens in dairy products. The assay targets the *femA*, *hly*, *ompA*, and *esxA* genes, respectively, with optimized primer/probe concentrations of 500 nM/400 nM and an annealing temperature of 58 °C. The established method demonstrated high specificity, with no cross-reactivity against common dairy-associated bacteria. The limits of detection (LoDs) ranged from 7.04 to 10.31 copies/reaction, with coefficients of variation (CVs) below 14% for intra-assay and 10% for inter-assay repeatability. Notably, the ddPCR assay detected a 25% co-contamination rate compared to 13% by qPCR among 120 dairy samples, suggesting higher sensitivity for low-abundance targets. This quadruplex ddPCR platform offers a rapid, sensitive, and high-throughput solution for food safety surveillance, particularly in high-risk dairy matrices such as infant formula.

## 1. Introduction

The complexity and detrimental impact of foodborne diseases, recognized as a worldwide public health concern, have become increasingly evident. According to estimates from the World Health Organization (WHO) concerning the global burden of foodborne illnesses, approximately 600 million individuals fall ill annually from consuming contaminated food, with around 420,000 deaths attributed to such incidents [1]. Among various foodborne pathogens, bacterial agents are the predominant cause of illness. Organisms such as *Staphylococcus aureus* (*S. aureus*), *Listeria monocytogenes* (*L. monocytogenes*), *C. sakazakii* (*Cronobacter sakazakii*), and *Mycobacterium bovis* (*M. bovis*) are of particular concern. Their distinct pathogenic mechanisms, marked environmental resilience, and elevated contamination risks associated with specific food matrices render them critical targets in food safety surveillance. These four bacterial pathogens are each associated with distinct food safety risks in different types of dairy products. *S. aureus* primarily causes acute food poisoning through the production of heat-stable enterotoxins [2]. *L. monocytogenes* poses an exceptionally high fatality risk for pregnant women, newborns, and immunocompromised individuals [3]. *C. sakazakii* represents a major threat in powdered infant formula, being capable of inducing severe neonatal meningitis and necrotizing enterocolitis [4]. *M. bovis*, a member of the *Mycobacterium tuberculosis* complex and a major zoonotic agent of bovine tuberculosis, is transmitted to humans primarily via the consumption of unpasteurized milk and dairy products, causing extrapulmonary tuberculosis with particular severity in immunocompromised individuals and children [5]. These four pathogenic bacteria share several common traits, including a low infectious dose, a brief incubation period, and the ability to cause severe clinical symptoms. Additionally, multidrug resistance has emerged in certain strains, posing serious challenges to conventional therapeutic approaches. As a result, the development of rapid, accurate, and high-throughput detection technologies is of great significance for enabling early warning of foodborne diseases and strengthening public health prevention and control measures.

Traditional methodologies for detecting foodborne pathogens [6,7,8] have largely depended on bacterial culture and isolation, a procedure that is both time-intensive and operationally cumbersome. With advancements in molecular biology, real-time quantitative PCR (qPCR) has emerged as a mainstream technique. However, qPCR remains susceptible to technical limitations, including fluorescence background interference from non-specific amplification or probe degradation, reduced sensitivity in complex food matrices due to PCR inhibitors, and its inherent reliance on external standard curves for quantification, which compromises absolute quantification accuracy and inter-laboratory reproducibility. As a third-generation nucleic acid amplification technology, droplet digital PCR (ddPCR) has recently demonstrated substantial potential in the detection of pathogenic microorganisms [9,10,11]. By partitioning the PCR mixture into tens of thousands to hundreds of thousands of micro-scale droplets for parallel amplification, ddPCR enables detection at the single-molecule level [9,12,13]. This approach eliminates the requirement for standard curves, a limitation inherent to qPCR, while also exhibiting enhanced tolerance to reaction inhibitors, thereby facilitating more accurate quantification in complex food matrices. Significant progress has been made in applying ddPCR technology for the detection of foodborne pathogens. For instance, a ddPCR assay targeting *L. monocytogenes* has been validated using artificially contaminated food samples, yielding results comparable to those obtained with traditional plate counting methods, thereby confirming its practical reliability [2]. Despite its considerable promise, current research on ddPCR remains predominantly focused on single- or dual-target detection.

Despite the considerable promise exhibited by ddPCR technology, the majority of current investigations remain confined to single- or dual-target detection [14,15,16]. Driven by the growing emphasis on precision detection and intelligent supervision, the field of foodborne pathogen testing is evolving toward high-throughput, automated, and portable approaches. In this study, a quadruplex ddPCR assay was established, which not only enhances laboratory testing efficiency but also holds broad applicability for quality control within food production enterprises, border inspection and quarantine operations, and clinical diagnosis of infections. Particularly in the surveillance of pathogenic bacteria in high-risk dairy products such as infant formula and yogurt, this technology offers a more robust level of assurance.

## 2. Materials and Methods

### 2.1. Bacterial Strains

*S. aureus* and *L. monocytogenes* were maintained in our laboratory, whereas *Salmonella enteritidis* (*S. enteritidis*), *Escherichia coli* (*E. coli*), *Bacillus cereus* (*B. cereus*), *Vibrio parahaemolyticus* (*V. parahaemolyticus*), *Vibrio vulnificus* (*V. vulnificus*), *C. sakazakii* and *M. bovis* were kindly donated by Zhejiang A&F University (ZAFU). All experiments involving these bacterial strains were conducted in a certified Biosafety Level 2 (BSL-2) laboratory.

### 2.2. Equipment and Reagents

The automated magnetic bead nucleic acid extraction system (GeneRotex 96) and bacterial genomic DNA extraction kit were purchased from Xi’an Tianlong Science and Technology Co., Ltd. (Xi’an, China). The LightCycler^®^ 480 real-time PCR system was obtained from Roche (Branchburg, NJ, USA). The QX600 droplet digital PCR system, ddPCR Supermix for Probes (No dUTP), droplet generation oil, and droplet analysis oil were purchased from Bio-Rad (Hercules, CA, USA). Premix Ex Taq™ (Probe qPCR) was obtained from Takara Bio (Kusatsu, Shiga, Japan). The Nanodrop spectrophotometer for nucleic acid quantification was purchased from Thermo Fisher Scientific (Waltham, MA, USA).

### 2.3. Preparation of Bacterial Cultures and Genomic DNA (gDNA) Extraction

The six bacterial strains, including *S. aureus*, *L. monocytogenes*, *S. enteritidis*, *E. coli*, *C. sakazakii*, and *M. bovis*, were individually inoculated into 10 mL nutrient broth medium (HKM, Guangdong, China) and incubated at 36 °C for 16–18 h. Bacterial DNA was extracted using the Bacterial genomic DNA extraction kit (TIANLONG, Xi’an, China) and finally eluted into 50 µL TE buffer. gDNA samples with concentrations of 10–100 ng/µL and purity ratios (A260/A280) of 1.7–1.9 were used for subsequent experiments.

### 2.4. Primers and Probes

To achieve specific detection of *S. aureus*, *L. monocytogenes*, *C. sakazakii*, and *M. bovis*, the *femA* gene sequence of *S. aureus* (Accession number: NC_017342.1), *hly* gene sequence of *L. monocytogenes* (Accession number: NC_003210), *ompA* gene sequence of *C. sakazakii* (Accession number: KU354286.1), and *esxA* gene sequence of *M. bovis* (Accession number: CP085582) from the GenBank database were used as target templates. Specific primers and probes were designed using Primer Express 3.0.1 software (ABI, Waltham, MA, USA), and the designed primer and probe sequences were subjected to specificity verification using the NCBI BLAST tool (version 2.16.0+). All primers and probes used in this method were synthesized by Sangon Biotech Co., Ltd. (Shanghai, China).

### 2.5. Construction and Quantification of Standard Plasmids

Based on the conserved gene sequences of each target bacteria in GenBank, amplification was performed using the primers listed in Table 1. The concentration was determined after dilution with ddH_2_O. The copy number concentration (copies/reaction) was calculated according to the following formula: [6.02 × 10^23^ × (plasmid concentration ng/µL × 10^−9^) × reaction volume (20 µL)]/[DNA length (bp) × 660]. The initial concentration was determined to be 1 × 108 copies/reaction. Serial dilutions were prepared with nuclease-free water at appropriate ratios and stored at −20 °C for subsequent experiments.

### 2.6. Specificity of the Quadruplex ddPCR

The specificity of the developed quadruplex ddPCR assay was assessed using genomic DNA extracted from common dairy-associated bacteria (*S. aureus*, *L. monocytogenes*, *S. enteritidis*, *E. coli*, *C. sakazakii*, and *M. bovis*) as templates. Nuclease-free water was employed as the negative control. The assay specificity was determined by detecting the presence or absence of positive droplet signals for each target pathogen.

### 2.7. Repeatability of the Quadruplex ddPCR

The intra-assay reproducibility was assessed by performing qPCR and ddPCR on serially diluted positive plasmids (10^1^–10^6^ copies/reaction) of *S. aureus*, *L. monocytogenes*, *C. sakazakii*, and *M. bovis*, with sixteen replicates per dilution. Coefficient of variation (Cv) was calculated for each dilution level. The CV calculation formula used is CV (%) = (SD/mean) × 100%. For inter-assay reproducibility, the plasmids were tested in four independent runs at different time points (four replicates per dilution), and Cv values were determined across the four batches.

### 2.8. Sensitivity of the Quadruplex ddPCR

To establish the sensitivity of the quadruplex ddPCR assay, standard plasmids containing target sequences of *S. aureus*, *L. monocytogenes*, *C. sakazakii*, and *M. bovis* were prepared at nine serial dilutions ranging from 1 × 10^5^ to 1 copy/reaction (1 × 10^5^, 1 × 10^4^, 1 × 10^3^, 1 × 10^2^, 10, 7.5, 5, 2.5, and 1 copy/reaction). Each dilution was subjected to ddPCR amplification in sixteen replicates. The mean concentration, standard deviation, and relative standard deviation were calculated for each dilution point. The limit of detection (LoD) was subsequently determined by probit regression analysis at 95% confidence interval based on 95% detection probability, thus comprehensively assessing the analytical sensitivity of the ddPCR assay.

### 2.9. Simulated and Dairy Sample Analysis

To evaluate the practical applicability of the developed quadruplex ddPCR assay, simulated contaminated samples were prepared by spiking serially diluted bacterial suspensions into dairy matrices. Briefly, *Staphylococcus aureus*, *Listeria monocytogenes*, *Cronobacter sakazakii*, and *Mycobacterium bovis* were individually inoculated into 10 mL of nutrient broth and incubated at 36 °C for 16–18 h to reach the logarithmic growth phase. After determining the bacterial concentrations, the cultures were serially diluted 10-fold with sterile PBS, and working suspensions with a concentration of 10^8^ copies/mL were obtained following quantification by ddPCR. For spiking, 1 mL of each diluted bacterial suspension was thoroughly mixed with 9 mL of dairy products (liquid milk and yogurt, provided by Zhejiang Liziyuan Food Co., Ltd., Jinhua, China) to achieve final concentrations ranging from 10^7^ to 10^1^ copies/g. Non-spiked dairy matrices served as negative controls. The spiked samples were incubated at 4 °C for 30 min to facilitate bacterial adsorption to the matrix components. Subsequently, nucleic acids were extracted using an automated magnetic bead-based extraction system (GeneRotex 96) and eluted into 50 μL of TE buffer; 2 μL of the extracted DNA was used as template for ddPCR detection. Each spiking level was tested in triplicate. The recovery rate was calculated as follows: Recovery (%) = (measured copies by ddPCR/theoretical spiked copies) × 100%. For evaluation of actual dairy samples, commercial dairy products obtained from Zhejiang Liziyuan Food Co., Ltd. were directly subjected to nucleic acid extraction and ddPCR analysis without artificial contamination. The detection results were used to preliminarily assess the practical performance and application value of the established quadruplex ddPCR assay.

## 3. Results

### 3.1. Optimization of the Quadruplex ddPCR Reaction System

**Figure 1:**
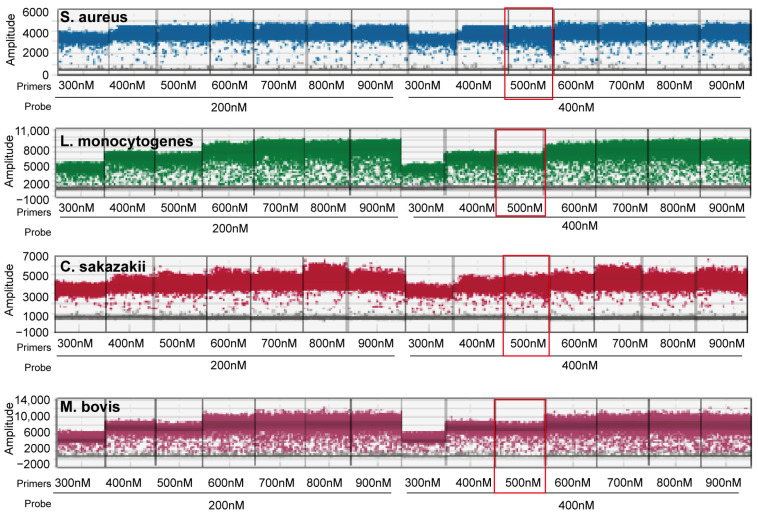
Optimization of concentration for the multiplex ddPCR system. The concentration ranges for primers and probes were optimized from 300 to 900 nM and 200 to 400 nM, respectively. Single-fluorescence channel results of multiplex ddPCR under different primer/probe combinations are shown, with positive signals indicated in color. The optimal primer/probe concentration is highlighted with a red box.

**Figure 2:**
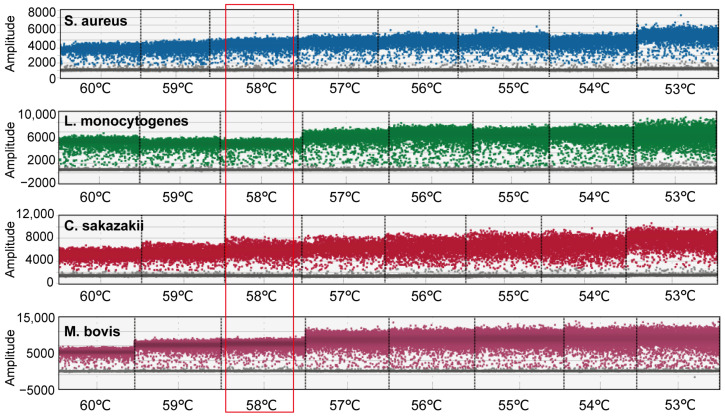
Optimization of the annealing temperature for the ddPCR system. Using positive plasmids of *S. aureus*, *L. monocytogenes*, *C. sakazakii*, and *M. bovis*, the annealing temperature for the ddPCR primers was optimized from 60 to 53 °C. By comparing the differences between negative and positive reactions and the number of droplets, the optimal annealing temperature was determined to be 58 °C (red box).

To optimize the quadruplex ddPCR reaction conditions, plasmid standards of *S. aureus*, *L. monocytogenes*, *C. sakazakii*, and *M. bovis* were used as templates. Gradient concentrations of forward and reverse primers were tested in the ddPCR reaction. The optimal primer/probe concentrations were determined based on the following criteria: (i) maximal positive droplet fluorescence amplitude to ensure sufficient signal-to-noise separation; (ii) minimal dispersion of positive droplet clusters to guarantee amplification consistency; and (iii) optimal economy to minimize reagent cost without compromising sensitivity. Based on the principles of maximum droplet amplitude and optimal economy, the optimal primer concentration for all four pathogens was determined to be 500 nmol/L, and the optimal probe concentration was 400 nmol/L for each target (Figure 1). The annealing temperature of ddPCR was subsequently optimized from 60 °C to 53 °C, and the quadruplex ddPCR results indicated that the optimal annealing temperature was 58 °C (Figure 2). Therefore, the optimized reaction system and thermal cycling program for the established quadruplex ddPCR assay were as follows: 5.0 μL Supermix, 1.0 μL primers (500 nM), 0.8 μL *S. aureus* probe (400 nM), 0.8 μL *L. monocytogenes* probe (400 nM), 0.8 μL *C. sakazakii* probe (400 nM), 0.8 μL *M. bovis* probe (400 nM), 8.8 μL nuclease-free water (ddH_2_O), and 2.0 μL template. The thermal cycling conditions were: 50 °C for 20 min; 95 °C for 10 min; 40 cycles of 94 °C for 30 s and 58 °C for 1 min; 98 °C for 10 min; and hold at 4 °C. The ramp rate for all heating and cooling steps was 2 °C/s.

### 3.2. Specificity of the Quadruplex ddPCR

**Figure 3:**
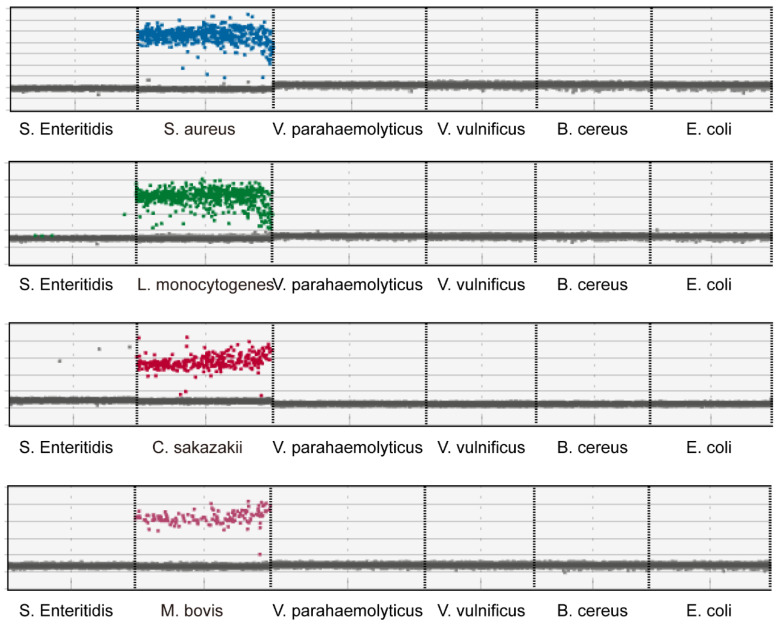
Specificity of the quadruplex ddPCR. The quadruplex ddPCR assay was performed to detect a mixture of the four target bacteria (*S. aureus*, *L. monocytogenes*, *C. sakazakii*, and *M. bovis*) and other common dairy pathogens (*Salmonella*, *E. coli*, *B. cereus*, *V. parahaemolyticus*, and *Vibrio vulnificus*), alongside a negative control (ddH2O). Positive droplets are colored blue (*S. aureus*), green (*L. monocytogenes*), red (*C. sakazakii*), and magenta (*M. bovis*). Negative droplets are colored grey.

Genomic DNA from *S. enteritidis*, *V. parahaemolyticus*, *V. vulnificus*, *B. cereus*, *S. aureus*, *E. coli*, *M. bovis*, *C. sakazakii*, and *L. monocytogenes*, along with a ddH_2_O negative control, was used as templates for ddPCR to assess specificity. Results showed that specific amplification occurred only in *S. aureus*, *L. monocytogenes*, *C. sakazakii*, and *M. bovis* whereas no positive fluorescence signals were observed in the other samples. This indicates high assay specificity without cross-reactivity (Figure 3).

### 3.3. Repeatability and Standard Curve

**Figure 4:**
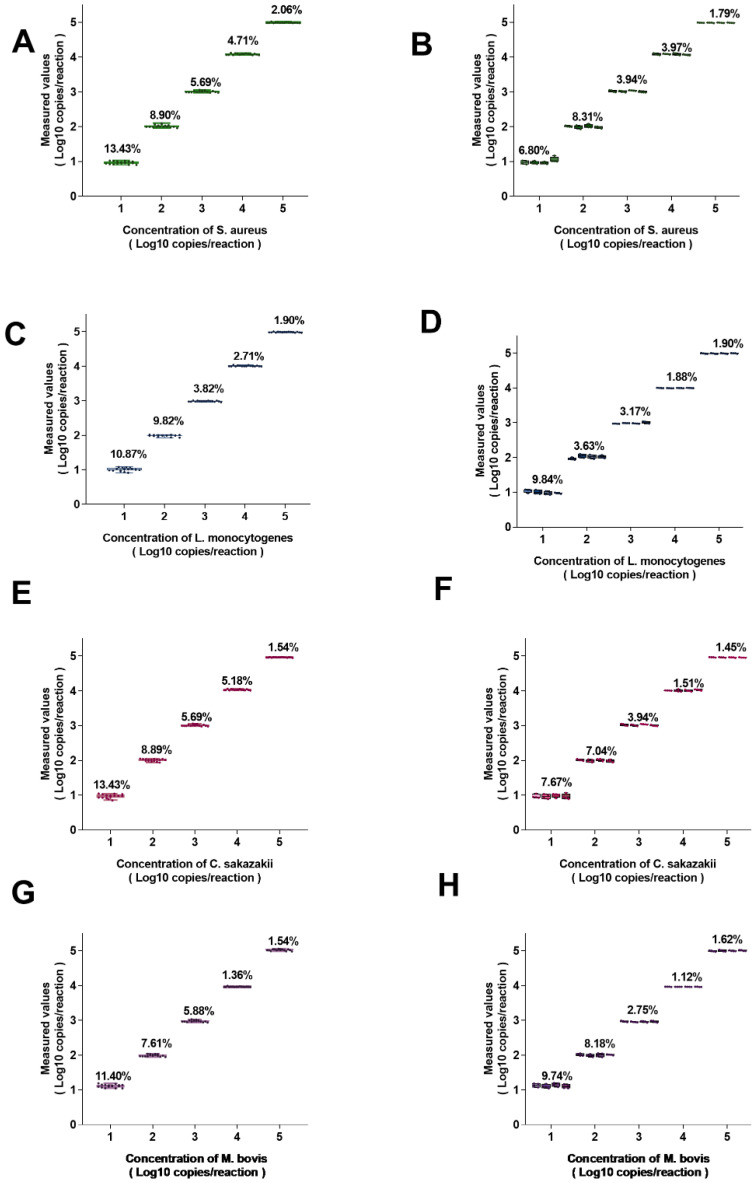
Intra- and inter-batch repeatability of the quadruplex digital PCR. The standard plasmids of (**A**,**B**) *S. aureus*, (**C**,**D**) *L. monocytogenes*, (**E**,**F**) *C. sakazakii*, and (**G**,**H**) *M. bovis*, with copy numbers varying from 10^1^ to 10^5^ copies/reaction, were detected by the established ddPCR. (**Left**): intra-batch repeatability with five concentrations (*n* = 16). (**Right**): inter-batch repeatability with five concentrations at four different time points (*n* = 4). X-axis: plasmid concentrations, Y-axis: measured values. Error bars represent the SD, and numerical values represent the CVs.

Positive plasmids of *S. aureus*, *L. monocytogenes*, *C. sakazakii*, and *M. bovis* at five concentrations (ranging from 10^1^ to 10^5^ copies/reaction) were used as templates for the ddPCR reaction. Sixteen replicates were performed for each dilution, and the corresponding coefficients of variation (CVs) were calculated. The results are shown in Figure 4: The intra-batch repeatability CVs for *S. aureus* ranged from 2.06% to 13.43% (Figure 4A); the intra-batch repeatability CVs for *L. monocytogenes* ranged from 1.90% to 10.87% (Figure 4C); the intra-batch repeatability CVs for *C. sakazakii* ranged from 1.54% to 13.43% (Figure 4E); the intra-batch repeatability CVs for *M. bovis* ranged from 1.54% to 11.40% (Figure 4G). All values were less than 14%. The aforementioned plasmids were tested at four different time points, with four replicates set for each dilution, to calculate the CVs across four batches of independent experiments. And the inter-batch repeatability CVs for *S. aureus* ranged from 1.79% to 6.80% (Figure 4B); the inter-batch repeatability CVs for *L. monocytogenes* ranged from 1.90% to 9.84% (Figure 4D); the inter-batch repeatability CVs for *C. sakazakii* ranged from 1.45% to 7.67% (Figure 4F); the inter-batch repeatability CVs for *M. bovis* ranged from 1.62% to 9.74% Figure 4H). All values were less than 10%. Within the detection range, the inter- and intra-batch repeatability CVs for the four pathogens were all below 14%, demonstrating that the method possesses high repeatability (Figure 4).

**Figure 5:**
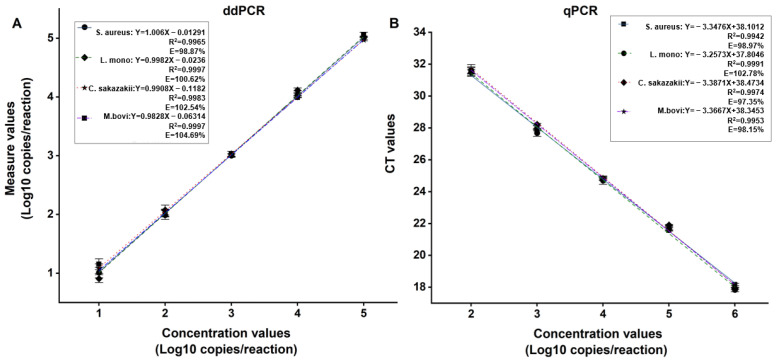
Standard curves of ddPCR and qPCR. (**A**) Standard curves of ddPCR. X-axis: Theoretical plasmid concentration; Y-axis: Actual measured concentration by ddPCR. (**B**) Standard curves of qPCR. X-axis: Theoretical plasmid concentration; Y-axis: CT values measured by qPCR. Data are presented as the mean ± standard deviation (SD) of 16 replicates. R^2^: Coefficient of determination. E: Amplification efficiency.

The standard curve was constructed based on the results of 16 replicates (Figure 5) and compared with the qPCR method. The results showed that the amplification efficiency (E) for the four target pathogens using the developed method ranged from 90% to 110%, with coefficients of determination (R^2^) greater than 0.996. These findings indicate a good fit between the observed and theoretical values, demonstrating the high stability of the assay. While the E and R^2^ values were comparable to those of the qPCR method, the latter failed to detect some of the 16 replicates at the concentration gradient of 10^1^ copies/reaction.

### 3.4. Sensitivity of the Quadruplex ddPCR

**Figure 6:**
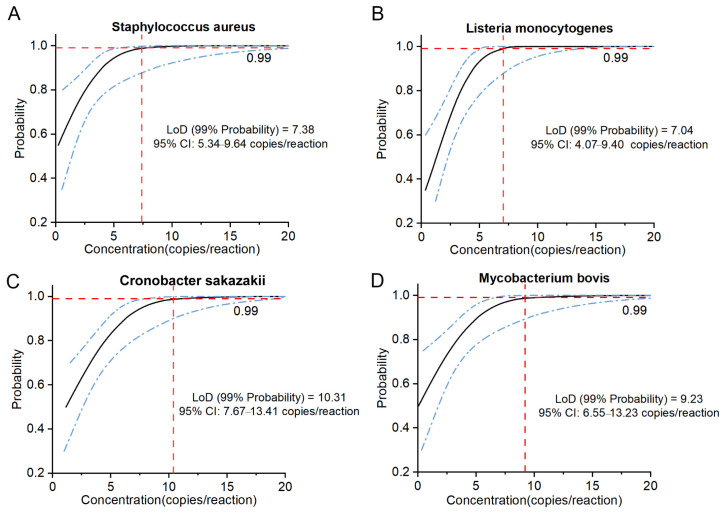
Probit analysis sigmoid curve reporting the limit of detection (LoD) of ddPCR. Positive plasmids corresponding to the following targets, with concentrations ranging from 1 × 10^0^ to 1 × 10^3^ copies/reaction, were used: (**A**) *S. aureus* (**B**) *L. monocytogenes* (**C**) *C. sakazakii* (**D**) *M. bovis*. Sixteen replicates were performed for each concentration. The limit of detection (LoD) was calculated within the 95% confidence interval (CI) according to the probit regression model, using a reproducible probability of 95% to assess the sensitivity of the assay. X-axis: Expected concentration (copies/reaction); Y-axis: Fraction of positive results in all parallel reactions performed. Black line: Probit curve. Blue dashed lines: 95% confidence intervals (95% CIs). Red cross lines: 95% probability of LoD.

The sensitivity assay was performed using standard plasmids with theoretical concentrations ranging from 1 × 10^0^ to 1 × 10^3^ copies/reaction. To determine the limits of detection (LoDs) of the ddPCR, probit regression analysis was conducted based on a 95% reproducibility probability (Figure 6). The limits of detection (LoDs) for *S. aureus*, *L. monocytogenes*, *C. sakazakii*, and *M. bovis* were determined to be 7.38 copies/reaction (95% CI: 5.34–9.64 copies/reaction), 7.04 copies/reaction (95% CI: 4.07–9.40 copies/reaction), 10.31 copies/reaction (95% CI: 7.67–13.41 copies/reaction), and 9.23 copies/reaction (95% CI: 6.55–13.23 copies/reaction), respectively.

### 3.5. Sample Detection by ddPCR and qPCR

**Figure 7:**
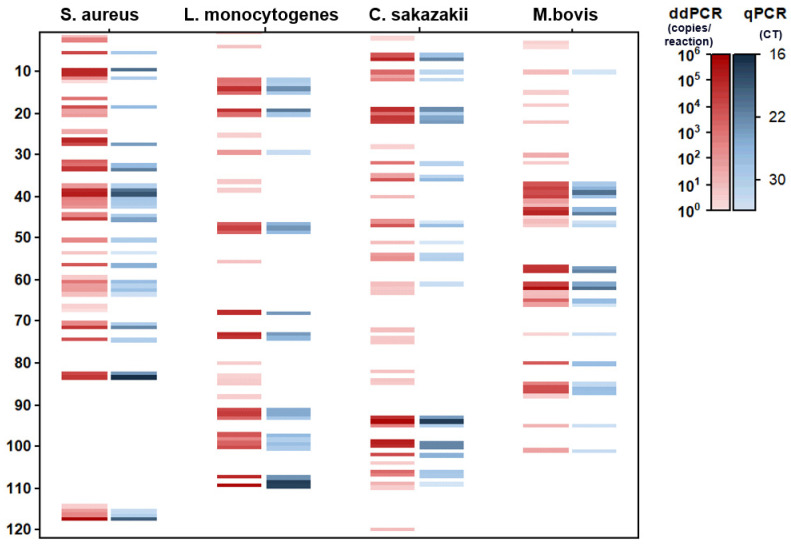
A total of 120 samples were analyzed by ddPCR (red) and qPCR (blue). The mean of the concentration (copies/reaction) or Cq value for each sample is scaled with red and blue colors, respectively.

**Figure 8:**
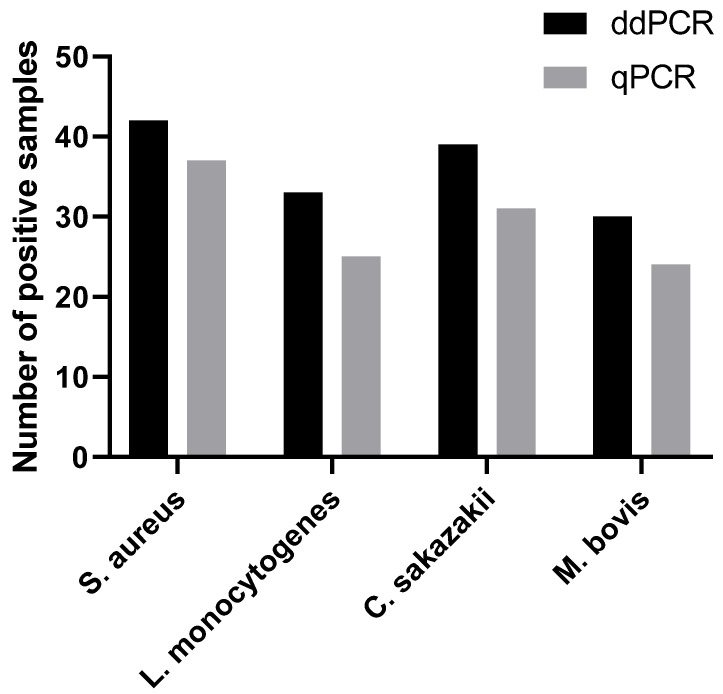
Comparison of positive detection for ddPCR and qPCR. Number of positive detections for each pathogen in 96 samples using ddPCR and qPCR.

A total of 120 dairy samples (including positive samples fortified with varying concentrations) were quantitatively analyzed using the established quadruplex ddPCR assay, and the results were compared with those obtained by the qPCR method (Figure 7). In the figure, color intensity denotes the bacterial concentration in the samples. The results showed that the ddPCR assay detected 42, 33, 39, and 30 positive samples for *S. aureus*, *L. monocytogenes*, *C. sakazakii*, and *M. bovis*, respectively, whereas the qPCR method detected 37, 25, 31, and 24 positive samples for the same targets. The risk of false-negative results arises when qPCR fails to detect the target bacteria at low concentrations (Figure 8). Notably, among the positive samples, the ddPCR assay identified 31 cases of co-contamination involving two or more pathogens, with the combination of *S. aureus* and *C. sakazakii* being the most prevalent. Cases involving simultaneous infection by four pathogens were even detected. These findings demonstrate that the positive detection rate of the ddPCR assay was significantly higher than that of the qPCR method.

To statistically evaluate the agreement between ddPCR and qPCR, Cohen’s kappa coefficient was calculated for each target pathogen based on the binary classification (positive/negative) of 120 dairy samples. The results showed substantial agreement between the two methods (Kappa > 0.75 for all targets), with ddPCR demonstrating higher sensitivity for low-abundance targets that were occasionally missed by qPCR (Ct > 35). Notably, among the 31 ddPCR-positive/qPCR-negative discordant samples, 26 (83.9%) exhibited concentrations below 10^2^ copies/reaction, consistent with the known limitation of qPCR in detecting near-limit targets. These statistical analyses, together with the co-contamination data presented in Table 2 (ddPCR: 25%, 31/120; qPCR: 13%, 16/120), collectively confirm that the quadruplex ddPCR assay offers superior diagnostic accuracy and sensitivity for food safety surveillance in complex dairy matrices.

## 4. Discussion

Foodborne diseases have emerged as one of the most pressing threats to public health [17,18,19,20]. The most prominent foodborne pathogens include *Salmonella* spp., *S. aureus*, and *L. monocytogenes*, among others. According to the Chinese national food safety standards, conventional microbiological detection methods are time-consuming and labor-intensive. Importantly, these methods are prone to the risk of false-negative results, particularly when the target bacteria are present at low concentrations.

Accurate and efficient detection of foodborne pathogenic microorganisms is a prerequisite for the effective prevention and control of foodborne diseases. In recent years, with the rapid development and deep integration of emerging technologies from multiple disciplines—including metabolomics, immunology, molecular biology, biosensing technology, laser technology, nanotechnology, and computer science—various novel rapid microbial detection methods have emerged, providing robust technical support for the screening and identification of foodborne bacterial pathogens [21,22,23]. Previous studies have reported the development of a rapid and simple quantitative identification method for *L. monocytogenes* in cheese using isothermal strand exchange amplification combined with surface-enhanced Raman spectroscopy [24]. Although the integration of isothermal nucleic acid amplification with Raman spectroscopic detection enables rapid nucleic acid testing without thermal cycling, this approach is designed exclusively for *L. monocytogenes* and lacks the capability for simultaneous detection of other foodborne pathogens. Furthermore, complex food matrices (e.g., fats and proteins present in cheese) may compromise the stability and reproducibility of the detection signal. Additionally, a quadruplex qPCR assay was developed for the detection and differentiation of *Campylobacter coli*, *Campylobacter fetus*, and *Campylobacter jejuni* in chicken and lamb [25]. Although this method enables simultaneous differentiation of three major *Campylobacter* species from other *Campylobacter* spp. in a single tube, it requires a qPCR instrument, which is unfavorable for on-site rapid detection and entails relatively high equipment costs. Currently, research based on droplet digital PCR (ddPCR) technology has achieved substantial progress, with a rapid increase in relevant publications [26,27,28,29,30,31]. The quadruplex ddPCR method established in this study demonstrates significant advantages over traditional qPCR in terms of detection sensitivity, accuracy of low-abundance quantification, and the ability to identify co-contamination, providing a high-throughput screening platform for dairy product safety monitoring. However, it should be cautiously noted that the standard plate counting method based on viable bacterial culture remains the irreplaceable gold standard for assessing bacterial viability and infectious activity, as its biological relevance cannot be matched by any molecular method. ddPCR detects DNA copy numbers and cannot distinguish between viable and dead bacteria, which constitutes an inherent limitation when evaluating actual food safety risks. Therefore, the appropriate role of this method should be as a rapid pre-screening tool preceding culture-based method. However, the vast majority of existing studies have focused on either qualitative identification or quantitative analysis of single target pathogens [2,32]. In contrast, multiplex ddPCR assays capable of simultaneous identification and quantification of multiple foodborne pathogenic bacteria remain relatively scarce, and the associated technical systems are yet to be fully established. The novelty of this study resides in the target panel redesign specific to the food safety risk spectrum of dairy products, substituting *Salmonella Typhi* and *B. cereus* [2] with *C. sakazakii* and *M. bovis*. This makes the detection panel more relevant to the practical surveillance demands of the dairy industry, alongside specific assay optimization for dairy matrices. This research landscape necessitates the construction of multiple independent detection workflows when facing complex samples potentially containing coexisting pathogenic threats in practical food testing applications, which not only significantly increases detection costs and time expenditure but also elevates the risk of cross-contamination during operation. Therefore, the development of efficient, sensitive, and cost-effective multiplex ddPCR platforms for the simultaneous screening and precise quantification of multiple foodborne pathogenic bacteria has become a critical technical bottleneck urgently requiring resolution in the field of food safety detection. The quadruplex ddPCR method developed in this study demonstrated lower limits of detection for the four bacterial targets, with *S. aureus*, *L. monocytogenes*, *C. sakazakii*, and *M. bovis* detecting at 7.38 copies/reaction, 7.04 copies/reaction, 10.31 copies/reaction, and 9.23 copies/reaction, respectively.

## 5. Conclusions

In summary, the quadruplex ddPCR assay developed in this study allows for the simultaneous and accurate quantification of four major foodborne pathogens—*S. aureus*, *L. monocytogenes*, *C. sakazakii*, and *M. bovis*—within a single reaction. Relative to conventional methods, this approach exhibits a reduced turnaround time, enhanced sensitivity (lower limits of detection), and superior reproducibility. Collectively, this method provides a high-throughput, rapid detection platform well-suited for food safety surveillance applications.

## Figures and Tables

**Table 1:** Primer and probe sequences.

Name	Sequence (5′→3′)	Amplification Product
*S. aureus*-F	AATCGCGGTCCAGTGA	132 bp
*S. aureus*-R	TGTAGCACAGGATCAAATCCT	
*S. aureus*-P	FAM-TGGCGAGATTACAGGTAATGCTGGT-BHQ1	
*L. monocytogenes*-F	AATCTGTCTCAGGTGATGTAGA	234 bp
*L. monocytogenes*-R	CCTCCAGAGTGATCGATGT	
*L. monocytogenes*-P	HEX-TCATCGACGGCAACCTCGGAGA-BHQ1	
*C. sakazakii*-F	AAACGGCGCTTTCAAAGC	224 bp
*C. sakazakii*-R	TATTCCAGACGGGTAGCGAT	
*C. sakazakii*-P	CY5-ACTGGGTTACCCGGTACCG-BHQ3	/
*M. bovis*-F	ACAGAGCAGCAGTGGAAT	219 bp
*M. bovis*-R	GCGTTGTTCAGCTCGGT	
*M. bovis*-P	ROX-ATCGAGGCCGCGGCAAG-BHQ2	/

**Table 2:** The results of Multiplex Bacterial Detection Rates by ddPCR and qPCR.

Target Pathogens	ddPCR Positive	qPCR Positive	Kappa (95% CI)	Concordance
*S. aureus*	42	37	0.78 (0.68–0.88)	92.5%
*L. monocytogenes*	33	25	0.76 (0.65–0.87)	90.8%
*C. sakazakii*	39	31	0.75 (0.64–0.86)	89.2%
*M. bovis*	30	24	0.79 (0.69–0.89)	93.3%
Co-contamination	31 (25%)	16 (13%)	/	/

## Data Availability

The original contributions presented in this study are included in the article. Further inquiries can be directed to the corresponding authors.

## Abbreviations

The following abbreviations are used in this manuscript:

ddPCR	Droplet Digital PCR
qPCR	Quantitative Real-Time PCR
LoD	Limit of Detection
CV	Coefficient of Variation
CI	Confidence Interval
